# Clinical trial of a low-cost external fixator for global surgery use

**DOI:** 10.1007/s00264-023-05807-9

**Published:** 2023-04-19

**Authors:** Giovanni Milandri, P. C. I. Wijesinghe, Dilshan Munidasa, Cinthuja Pathmanathan, Mehdi Saeidi, Anthony M. J. Bull, Pujitha Silva

**Affiliations:** 1 Department of Bioengineering, Imperial College London, London, UK; 2 National Hospital of Sri Lanka, Colombo, Sri Lanka; 3 Faculty of Medicine, University of Colombo, Colombo, Sri Lanka; 4 University of Moratuwa, Moratuwa, Sri Lanka

**Keywords:** Trauma, Orthopaedics, External fixator, Global surgery, Damage control orthopaedics

## Abstract

**Purpose:**

A low-cost modular external fixator for the lower limb has been developed for global surgery use. The purpose of this study is to assess outcome measures in the first clinical use of the device.

**Methods:**

A prospective cohort study was conducted with patients recruited in two trauma hospitals. Initial clinical procedure data were collected, and patients were followed up every two weeks until 12 weeks or definitive fixation. Follow-up assessed infection, stability, and radiographic outcomes. In addition, patient-reported outcomes and surgeons’ feedback on device usability were collected by questionnaires.

**Results:**

The external fixator was used on 17 patients. Ten were mono-lateral, five were joint spanning, and two were delta configuration. One patient had a pin site infection at 12-week follow-up. All were stable when tested mechanically and using radiographic assessment, and 53% were converted to definitive fixation.

**Conclusion:**

The low-cost external fixator developed is appropriate for use in global surgery trauma centres with good clinical outcomes.

**Prospective trial registration number and date:**

SLCTR/2021/025 (06 Sep 2021).

## Introduction

Globally, there is an acute need for surgical devices to treat fractures due to, for example, the dramatic increase in road traffic injuries [1]. In Sri Lanka, the number of road traffic injuries has been steadily increasing, with the rate doubling since the mid-1960s and is currently approximately 120 per 100,000 population [2]. This trend has been mirrored in many countries globally, with the global increase happening predominantly in low- and middle-income countries (LMICs), which bear 90% of the burden of these injuries [3].

Global surgery can be defined as ‘the enterprise of providing improved and equitable surgical care to the world’s population, with its core tenets as the issues of need, access and quality’ [4]. In global surgery, treatment of open fractures by external fixation has been deemed part of essential surgical care by the Lancet Commission on Global Surgery (2014) and has been selected as one of the three *Bellwether Procedures* to indicate the level of access to surgery [5].

External fixation is recommended for all open fractures (Gustilo-Anderson grade II and above) of any type when definitive stabilisation and immediate wound cover are not carried out at the time of primary debridement [6]. It has become the standard of care for temporary fixation of open fractures in global surgery, including disaster relief [7], conflict, and routine care settings, in urban and field hospital settings, and has also been used as definitive fixation [8]. A survey to identify core surgical competencies for humanitarian response found that 80% of 147 surgeons agreed or strongly agree that external fixation should be included [9].

Temporary external fixation is also a cornerstone of damage control orthopaedics (DCO) [10–12], where orthoplastic surgical teams yield better outcomes than orthopaedic teams alone in LMIC settings [13]. In the disaster response by Médecins Sans Frontières to the 2010 Haiti earthquake where there was an acute lack of orthopaedic surgeons, doctors of non-trauma specialties were successfully taught the external fixation technique using the GexFix fixator kit (Carouge, Switzerland). This device was also successfully used in the Democratic Republic of Congo, dramatically reducing the amputation rate from 100% to approximately 20% between 2007 and 2013 [14]. In Afghanistan in 2012, this same device reduced the amputation rate from approximately 50% initially to approximately 20% within three months and stayed at that point for the remainder of the monitoring period [14].

Despite this success, published complications of external fixators include pin loosening, pin site infection (PSI), nonunion/malunion, periprosthetic fractures, and osteomyelitis [15]. In addition to complications, the lack of access to fixators in the LMIC setting and the lack of timely access in conflict zones, where case numbers suddenly increase, has resulted in improvisation using wood [16] or pin-in-plaster techniques [17]. Other appropriate locally manufactured device designs have been developed and used [18–22]. These devices have shown promise in short-term follow-up, reuse of devices to reduce costs, and removing the common barrier to access of initial device cost.

However, these devices have not achieved widespread adoption and there remains the need for an appropriate fixator that is easy to use or reuse, can be manufactured using readily available material and skillset using conventional workshop equipment, and provides stiffness similar to commercial fixators. A recently developed appropriate external fixator is a frugal value innovation [23] that responds to this need. The fixator design has been tested thoroughly including a cadaver study with eight specimens showing similar stiffness to Hoffman® III fixator [24]. The aim of this study is to benchmark clinical outcomes of this low-cost external fixator.

## Materials and methods

The appropriate external fixator which was used is modular with large (rod) clamps, and small (pin) aluminium clamps compatible with 5 mm Schanz pins, and stainless-steel rods of various lengths to suit the application. The design drawings of the fixator are open source and are available online.^1^ In order to ensure consistent quality of the device, the clamps were manufactured using a computer numerical control machines.

Two surgeons (CW, DM) applied the appropriate external fixator at a level one trauma hospital and a regional main trauma hospital during 2021 and 2022 over 11 months on 17 patients. Approval was granted by the Ethics Committee of the university faculty of medicine, with permission from hospital directors, and the trial was prospectively registered with the national clinical trial registry (Date: 06 Sep 2021/No: SLCTR/202I/025) and referenced in the WHO International Clinical Trials Registry Platform. After acute management, informed consent was obtained from the patient, or their family if the patient was not conscious at admission. In all cases, consent included publication of anonymised images of the treatment of the lower limb for research purposes.

Patient management, wound debridement and external fixation were done to hospital standard practice according to the 2020 guidelines of the British Association of Plastic Reconstructive and Aesthetic Surgeons (BAPRAS) [6]. The fracture was assessed using both the Mangled Extremity Severity Score (MESS) [25] and the Gustilo-Anderson Classification of injury. A monolateral configuration was used for mid-diaphyseal fractures, with a short rod-to-limb distance for maximum stiffness and a lower risk of neurovascular injury compared to bilateral fixation. This was extended by the surgeons with ethical approval to include joint spanning or delta configurations for peri-articular or ankle fractures, respectively. Pin count and configuration were decided by the treating orthopaedic surgeon. Commercially available sterile Schanz pins were used, hydroxyapatite-coated wherever available. Thermal damage from drilling was minimised wherever possible by cooling with saline and using stop-start drilling. Pin site infection (PSI) control included daily pin site cleaning with surgical spirit and applying a sterile dry dressing to each pin site. After  one week, pin sites were cleaned daily with surgical spirit, but no dressing was used. Discharge criteria were standard for the hospital. Once removed the device was cleaned and sterilised and reused where possible.

Follow-up was performed at two, four, six, eight and 12 weeks, even if definitive fixation was achieved. Follow-up testing included clinical data of the presence of pin site infection and evidence of pin loosening. PSI was defined as pain or inflammation at the pin site accompanied by discharge which is either positive to bacterial culture or responded to a course of antibiotics. PSI grading was conducted using the Checketts-Otterburn classification [26]. The stability of the construct was assessed by whether the fixator could be lifted up freely or if it needed support, a method commonly used by surgeons. Follow-up radiology assessed secondary loss of fracture reduction and evidence of callus formation. Evidence for osteomyelitis was assessed by the attending clinician, and was monitored using MRI if necessary in the short term, and radiographs for longer term. For this full period, weight-bearing was contra-indicated. Progression of soft tissue healing was assessed by whether the fracture was suitable for definitive fixation, or where it met the criterion of radiographic evidence of bone union in which case it was removed. For all the above measures, the percentages which are reported below were calculated by the remaining patients using temporary external fixation at that time point.

Surgeon feedback was obtained using a self-administered questionnaire to assess the appropriate external fixator’s ease of use, applicability, and problems faced during surgery. Patients also reported their experience of the device through semi-structured interviews and were encouraged to give suggestions on how to improve the device. Descriptive statistics were calculated using SPSS 20.

## Results

The seventeen participants were young, predominantly male (88%) and spanned the range of injury severity (Table 1). Pin site infection and pin loosening occurred in one patient at two weeks, all constructs were stable, and there was no sign of osteomyelitis or secondary loss of reduction (Table 2). No patients dropped out of the study.

**Table 1:** Patient injury and demographic characteristics

Characteristic	Result
Number of patients	17
Sex	2 (12%) female, 15 (88%) male
Mean age	38.8 years (SD15.4, range: 19–70 years)
Cause of injury	Road traffic accident – 14 (82%); workplace injury — 1 (6%); other reasons — 2 (12%)
Fractured bone	13 (76%) tibia (including 2 distal tibia), 4 (24%) femur
Fracture site	10 (59%) diaphyseal, 7 (41%) metaphyseal
MESS score < 7	14 (81%)
Injury severity (Gustilo-Anderson classification)	1 (3 patients), 2 (5 patients), 3A (1 patient), 3B (4 patients), 3C (1 patient), and closed (3 patients)
Admission timing	11 (65%) within 24h
Fixator configuration	10 (59%) unilateral uniplanar configuration, 5 (29%) spanning configuration, 2 (12%) delta configuration
Type of soft tissue closure	6 (35%) primary closure, 5 (29%) secondary closure, 1 (6%) split skin graft, 3 (18%) reconstructive procedure, 1 (6%) wound dressing, 1 (6%) awaiting amputation due to vascular injury

**Table 2:** Clinical and radiographic outcomes at each follow-up assessment, expressed as percentage (%) of those still in use. No patients dropped out of the study

Outcome	2 weeks	4 weeks	6 weeks	8 weeks	12 weeks
Number of patients still using temporary external fixation	17	11	10	7	6
Pin site infection	1 (6%)	1 (9%)	1 (10%)	0 (0%)	0 (0%)
Pin site loosening	1 (6%)	0%	0%	0%	0%
Construct stability	17 (100%)	11 (100%)	10 (100%)	7 (100%)	6 (100%)
Radiological evidence of secondary loss of reduction	0%	0%	0%	0%	0%
Evidence of osteomyelitis	0%	0%	0%	0%	0%

**Fig. 1:**
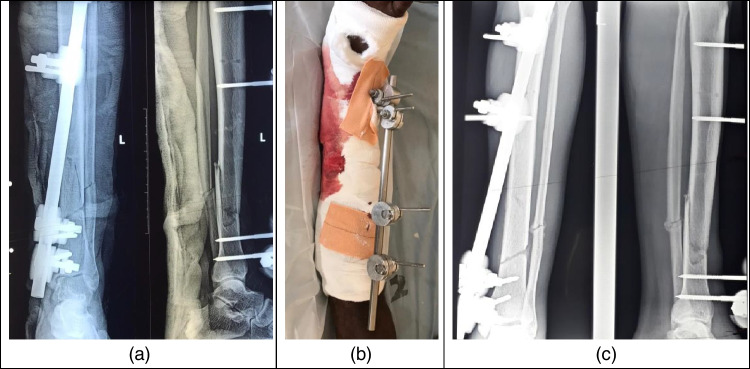
Case study of the external fixator’s unilateral uniplanar configuration. **a** Initial radiographs. **b** External view of initial fixation. **c** Radiographs at 2 weeks

**Fig. 2:**
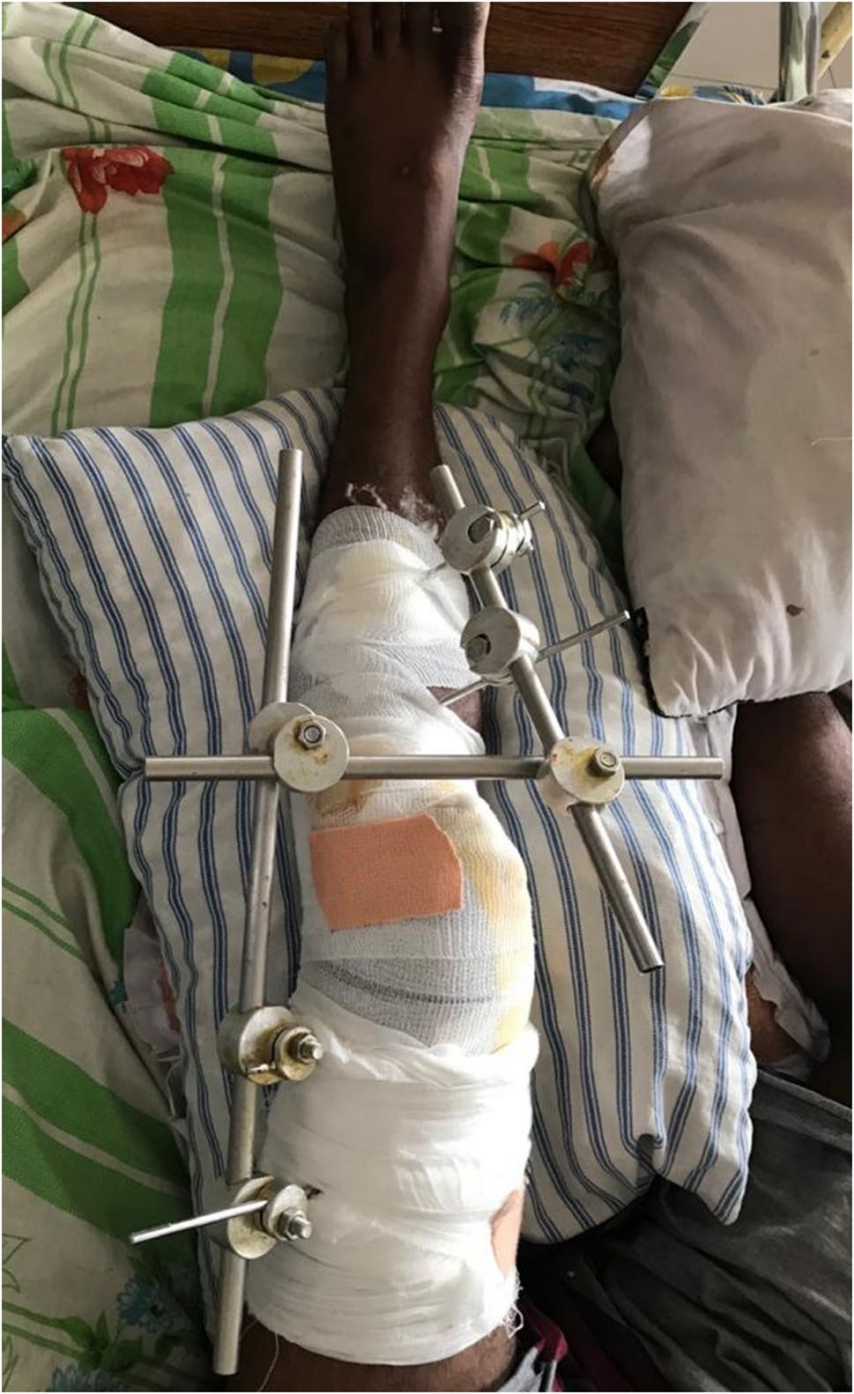
Joint spanning configuration

**Fig. 3:**
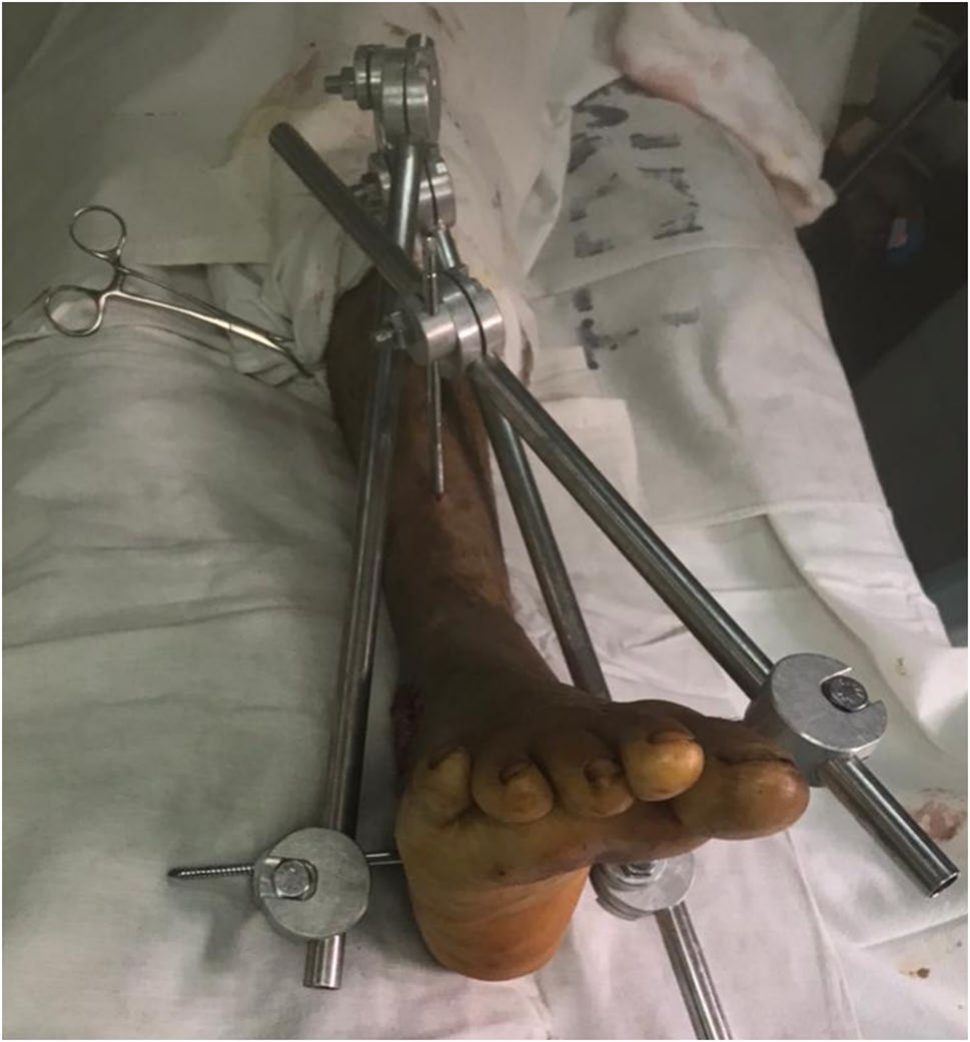
Delta configuration

Figures 1, 2, and 3 are case studies of unilateral uniplanar, joint spanning, and delta configurations, respectively.

Both surgeons and six patients provided feedback (Table 3). Overall surgeon feedback on maintaining fracture reduction, stability of the external fixator, and setting up the construct was ‘very good’. The following positive points of the fixator were highlighted:

- With the current design, it is possible to construct unilateral multiplanar configuration
- With the current design, it is possible to construct modular and spanning configurations

**Table 3:** Surgeon (*n* = 2) and patient (*n* = 6) feedback

	Questions	Response	Number
Surgeon responses	Time taken to set up the external fixator compared to the commonly used reference device (AO fixator)	Equal	1
		Less	1
	Freedom of pin placement	Very good	1
		Satisfactory	1
	Conformity of clamps, rods, and Schanz screws	Very good	2
	Ability to stabilise the fracture reduction	Very good	1
		Satisfactory	1
	Are you confident fracture reduction will be maintained?	Yes	2
	Did you or your assistant experience any injuries while setting up the fixator?	No	2
	Was there a risk of injury handling the components?	No risk	2
	Overall impression of the appropriate external fixator	Very good	2
Patient responses	Are you able to move with ease?	Yes	2
		No	4
	Are you able to walk with crutches?	No	6
	Toileting	Not difficult	4
		Difficult	2
	Are you able to sit?	Yes, with ease	6
	Are you able to weight bear?	No	6
	Are you able to bathe?	Yes, with ease	4
		Yes, with difficulty	2
	Are you able to roll over?	Yes, with ease	4
		Yes, with difficulty	2
	Is the external fixator too heavy?	No	4
		Yes	2

The following design recommendations were made:

- Reduce clamp size
- Roughen the clamp surfaces for better friction and stiffness
- Increase screw (bolt) length in clamp-to-clamp application
- During spanning external fixator rod-to-rod clamping, the length of clamp connecting screw would be improved by being marginally lengthened.

Four out of six patients who gave feedback mentioned that movements are not inconvenient with the external fixator, none of them weight bear through the external fixator leg and two patients felt that the external fixator was too heavy. However, there were variations in responses for other functional activities with the external fixator.

One patient with a joint spanning configuration felt that the fixator alignment on application should be improved. One patient suggested a reduction in external fixator length. One patient highlighted that pain was a problem and wanted less restriction in turning.

## Discussion

The results of this clinical trial of the external fixator show that the device can successfully be used in a lower limb long bone fracture stabilisation, as definitive fixation for a diversity of configurations and soft tissue closures. Thus, it can provide a low-cost solution for hospitals lacking equipment of this type.

Regarding stability results, the device showed excellent radiographic and clinically assessed stability up to removal in all patients. Thus, the mechanical stiffness and load capacity of the device are sufficient for the use indicated.

Pin site infection was only encountered in one patient. The rate of pin site infection below 10% is low, and below the published mean rate in LMICs of 18% [26] compared to other external fixators of any type. Pin loosening was only seen for one patient (6%) at the first follow-up visit (2 weeks).

This trial included cases of joint spanning and delta configurations. This shows that the device is sufficiently modular to be successfully used in these other configurations, based on the surgeon’s existing knowledge and experience.

In the survey responses, surgeons were satisfied with the performance and usability of the device, both rating the overall impression as ‘very good’. On the other questionnaire domains, they rated the device to be ‘very good’ or ‘satisfactory’ and the device took either an equal or less amount of time to set up than the reference device.

Patient feedback was positive or mixed, but there was no comparison with other devices. None of the patients reported walking on the device, and walking was contraindicated in this study. However, walking while wearing the external fixator has been shown to improve healing potential of the fracture site [27, 28], and should be investigated in future studies.

The fixator was used in two tertiary care hospitals in Sri Lanka. Both institutes are final referral centres for polytrauma patients. External fixation is one of the commonest orthopaedic trauma services provided in both centres. The main mechanism of injury is high velocity due to motor vehicle and occupational accidents. Approximately one to three external fixations are performed daily for these injuries, which are most often open fractures or periarticular fractures.

In this study, the external fixator was successfully sterilised and reused. This should be seen to be the likely default for fixators used in LMICs, and is generally recommended [29, 30], although some researchers have discouraged the practice [31]. Further validation research is required to establish what the limits of reuse are in terms of durability and sterilisation. Notwithstanding these points, reuse contains cost [32] and ensures a higher availability of the device.

Regarding costs, the factory gate cost of the device was approximately GBP100 when manufactured using CNC machining in small batches in the UK. While local, manual manufacturing in the country of use and higher scale manufacturing may have the potential to reduce this cost, estimates received to date from suppliers in Sri Lanka indicate that this would be a reasonable expectation of the factory gate cost for a sustainable business model. This would result in an approximate sale price of GBP125-250 (USD150-300). This includes allowance for medical device quality assurance, local regulatory approval, distribution, stock-keeping, customer support, and other business functions. Thus, the total price is approximately 1/10 of the sales price of a commercial Hoffmann fixator at current prices. Sterilisation and reuse of the devices would further reduce the cost per use.

The study has a number of limitations, including the inability to definitively assess union/mal-union at 12 weeks’ follow-up. Some definitions of non-union refer to lack of union of up to six months, or even up to a year [33]. Additionally, this study was undertaken without a group tested with a comparator device. In this public setting, generally, there is a lack of external fixators; thus, there was not a suitable alternative to compare with, nor was there an established data set for retrospective comparison. A third limitation is that the joint spanning and delta configurations were used only in few cases and can be seen as pilot uses only of these types.

Although there have been reports in the literature of other low-cost external fixators, this work presents some new features. First, this device is the first to have an open source published design, which allows it to be rapidly manufactured (surge capacity) near the point of use for humanitarian, conflict, and resource-constrained settings. Second, it is designed for local manufacture using stock materials and commonly available tools. Finally, this is the first external fixator for low-cost clinical use we have found that has published mechanical and cadaver testing results, ensuring that those who use it can be confident of its performance. Thus, the clinical trial results reported in this manuscript represent an additional component of the evidence for its effectiveness in clinical use.

In conclusion, this trial has demonstrated that the device can be effectively used clinically for use in a global surgery trauma setting for the outcomes set out, requires minimal additional training, and can be sterilised and reused. Thus, this device is an effective and appropriate design for use in civilian settings and it is likely that it can additionally be used in humanitarian and conflict settings.

## Data Availability

None

## Footnotes

1 https://www.imperial.ac.uk/external-fixator/

## Declarations

### Disclaimer

The views expressed are those of the author(s) and not necessarily those of the NIHR or the UK government.
